# Delusion of Pregnancy in a 64-Year-Old Male: A Case Report

**DOI:** 10.7759/cureus.84478

**Published:** 2025-05-20

**Authors:** Vikash Mehan, Maximilian Wehner, Kendahl Oberdorfer, Zaira Khalid

**Affiliations:** 1 Psychiatry, Wayne State University School of Medicine, Detroit, USA; 2 Psychiatry, Henry Ford Health System, Detroit, USA

**Keywords:** delusion of pregnancy, geriatric psychiatry, homosexual, male delusion of pregnancy, psychiatry disorders, schizophrenia and other psychotic disorders, sexual activity, sexual distress, somatic delusion, urine pregnancy test

## Abstract

This case describes an instance of delusion of pregnancy in an older male. This patient has a past history of schizophrenia and reported the delusion soon after engaging in a consensual sexual act with another male for the first time. Delusion of pregnancy is a rare disorder, especially among males and older adults. Along with the use of antipsychotic medications, we discuss the approach of using a pregnancy test in a male to aid in reality orientation and dispel the delusion of pregnancy.

## Introduction

This report discusses a case of delusion of pregnancy in a 64-year-old male with a past medical history of schizophrenia. Delusion of pregnancy represents a fixed false belief of being pregnant despite evidence showing the absence of pregnancy [1]. In the absence of other symptoms, the Diagnostic and Statistical Manual of Mental Disorders, Fifth Edition (DSM-5), would classify this condition as "delusional disorder, somatic type with bizarre content." However, in this case, with a pre-existing diagnosis of schizophrenia, this delusion would be classified under a diagnosis of "schizophrenia" [2]. Few case reports have been published that report an incidence in males, and the exact mechanism underlying the disorder is unknown. A study by Bera and Sarkar found that out of 84 reported cases, 76.2% of the cases were in females and 23.8% were in males. Further, among all cases, only 18% were in individuals over the age of 60 years [3]. The emergence of delusion of pregnancy in men has been strongly associated with impaired cerebral function, a history of male sexual abuse, and contact with pregnant women [4]. 

The condition must be differentiated from couvade syndrome, which is more commonly found in males and not formally listed in the DSM-5. Couvade syndrome is a psychosomatic disorder that can cause an expectant father to experience physical and psychological symptoms similar to those of their pregnant partner. Couvade syndrome has a variable incidence ranging from 11% to 65% and involves symptoms including appetite variations, nausea, insomnia, and weight gain [5]. The condition is also similar to pseudocyesis, which is a persistent false belief of being pregnant, but differs in that pseudocyesis is associated with objective signs and symptoms of pregnancy [2]. Pseudocyesis has the highest incidence in developing countries that strongly emphasize fertility and childbearing [6]. Delusions of pregnancy have also been found to be related to organic medical and surgical comorbidities, which must be ruled out before being fully attributed to psychiatric delusions [7].

This case report aims to expand clinical awareness of delusion of pregnancy by presenting a rare instance in a 64-year-old male with schizophrenia, emerging shortly after a same-sex encounter. While delusion of pregnancy is more commonly documented in younger females, this case highlights its atypical presentation in an older male without gender dysphoria. It also illustrates the novel use of a urine pregnancy test in a male patient as a tool for reality testing, which ultimately contributed to the resolution of the delusion.

## Case presentation

We present the case of a 64-year-old cis-White male with a documented past history of schizophrenia, type 2 diabetes, hypertension, and hyperlipidemia who initially presented to the emergency department via emergency medical services (EMS) after being found asleep outside a fire station and having a disorganized thought process. Laboratory values on admission were notable for hyponatremia, hypokalemia, hypochloremia, hypomagnesemia, elevated creatine phosphokinase (CPK), and elevated aspartate aminotransferase (AST) (Table 1). His urine drug screen was negative. Home medications, with which he was non-compliant, included olanzapine 20 mg twice a day, quetiapine 400 mg at bedtime, and sertraline 50 mg daily. 

**Table 1:** Pertinent abnormal laboratory values at admission CPK: creatine phosphokinase; AST: aspartate aminotransferase

Parameter	Patient Value	Normal Limits
Sodium	125 mmol/L	136 to 145 mmol/L
Potassium	3.3 mmol/L	3.5 to 5 mmol/L
Chloride	90 mmol/L	98 to 106 mmol/L
Magnesium	1.6 mg/dL	1.6 to 2.6 mg/dL
CPK	2109 units/L	55 to 170 units/L
AST	73 units/L	10 to 40 units/L

In the emergency department, he received imaging related to his injuries. A CT head, chest X-ray, and shoulder X-ray were unremarkable. Nephrology was involved in managing the hyponatremia. Fluid restriction was ordered with subsequent improvement in serum sodium levels. Other electrolyte abnormalities were also medically corrected. While being managed by the nephrology service, the patient reported delusions of being pregnant. Consultation-liaison psychiatry was involved for management of the delusions and schizophrenia. In the interview with psychiatry, while still admitted to the nephrology service, the patient demonstrated loose associations with delusional content, which was grandiose in nature. The patient was subsequently deemed medically stable and transferred to the psychiatric hospital, where he continued to endorse delusions even after the resolution of his initial metabolic abnormalities. Of note, the patient denied substance use, and his urine drug screen was negative for amphetamine, barbiturates, benzodiazepines, cannabinoids, cocaine, opiates, phencyclidine, and tricyclic antidepressants.

The patient identified as a heterosexual male; however, he reported interest in being sexual with a male for most of his life. He described a time when he was younger when he engaged in non-penetrative sexual activity with another male; however, he did not have penetrative intercourse with a male until the days leading up to hospitalization, which he states led to pregnancy. The patient had been married three times in heterosexual relationships and had three children. 

At the psychiatric hospital, he continued to express his concern about being pregnant. At the facility, he was found to be irritable and suspicious of those around him. He was also assessed to have an unpredictable and disorganized thought pattern. He was observed to be responding to internal stimuli and reported hearing voices calling his name.

He endorsed that he was “bleeding and spotting” due to pregnancy. He believed he had a protruding abdomen due to the growing fetus, resulting in an increased appetite and oral intake. The patient endorsed being able to feel fetal movements as well. When asked how he would deliver the child, he made a vague statement that he did not have enough blood flow to his genitals, so he would need a cesarean section. The idea of having and raising a child caused distress and anxiety for the patient, as he frequently made remarks about ensuring that the other parent of the child would have to pay child support. 

The patient was treated with antipsychotics for disorganization and delusions; a trial of olanzapine orally at a maximum tolerated dose of 20 mg was ineffective, and the patient was transitioned to fluphenazine 5 mg twice daily with improved flow of thought process but no improvement of delusions. 

After consultation with the patient and with his consent, a urine pregnancy test was obtained to give the patient objective evidence contrary to his delusion about being pregnant. When given the results of a negative pregnancy test, the patient was surprised but relieved. Another notable finding was the reduction of as-needed psychotropics required for agitation after receiving the test results. In the days leading up to discharge, the patient no longer endorsed delusions of pregnancy.

## Discussion

We provide here a unique case of a delusion of pregnancy in a geriatric male. This case is a relative rarity, as the vast majority of delusions of pregnancy recorded in the literature occur in reproductive-aged females. A systematic review from 2015 of 84 case reports of delusions of pregnancy found that only 23.8% of total cases were in males, and only 17.9% of total cases occurred in those over the age of 60 [3]. 

There have been reported cases of delusions of pregnancy in men after homosexual encounters. In one reported case of a 70-year-old male with a delusion of pregnancy after a homosexual encounter, the patient reported a belief that they were turning into a female and showing signs of breast growth, and as a result, limited their food intake [8]. In another case of a 24-year-old man who had twice engaged in homosexual acts as the passive partner, he believed that he had become pregnant. He experienced anxiety about the delusional pregnancy, with a belief that he could feel the movements of the fetus and with a desire to terminate the pregnancy. It was reported that he had recently read a magazine story about a man who had become pregnant [9]. Similar to other reports, our patient also endorsed physical symptoms of a pregnancy, a protruding abdomen, increased appetite, and fetal movements. What made this patient's encounter unique was that he did not report any gender dysphoria, had no desire to terminate the pregnancy, and had no trouble believing a biological male could be pregnant. Further, any psychological distress he may have felt as a result of the believed pregnancy was channeled only into a preoccupation that the man who he believed impregnated him would be forced to pay child support.

In the systematic review of 84 cases of delusion of pregnancy, perception of fetal movement was noted in 45.2% of cases, and coenaesthopathological processes, abnormal somatic experiences such as distorted proprioceptive or visceral sensations, were implicated in 20.2% of cases [3]. Our patient exhibited similar somatic preoccupations, including a protruding abdomen and the subjective experience of fetal movement. His medical history was notable for an open ileocolectomy for an appendiceal mucocele six years prior, as well as multiple emergency visits over the past four years for abdominal pain, diarrhea, constipation, and occasional blood in the stool. During psychiatric evaluation, he remained intensely focused on abdominal sensations. A 2020 review of 140 cases of delusion of pregnancy further supports the significance of organic factors, identifying that in 23 cases, underlying organic medical or surgical conditions, such as gallstones or abdominal masses, contributed to the delusion, with resolution in 10 out of 15 cases that underwent medical intervention [7]. Although no active pathology was found during the workup of our patient, his somatic history remains relevant. According to the cognitive model of delusions, fixed false beliefs may arise as attempts to rationalize abnormal bodily experiences [10]. In this case, the patient’s reported abdominal symptoms, potentially arising from subclinical gastrointestinal irregularities, combined with recent homosexual intercourse, may have coalesced into a delusional conviction of pregnancy, perceived by him as a coherent and meaningful explanation for his physical and emotional experience.

A unique feature of our case not found elsewhere in our literature review was the use of a urine pregnancy test to assist in reality testing. In a 1994 paper by Chadwick and Lowe using a cognitive approach to modify delusions, the authors found that reality testing following a verbal challenge to a delusion worked most strongly in being able to modify the delusion [10]. Our team took a similar approach in working to dissolve the patient’s delusion. During interviews with the patient, we broached the topic of the possibility of pregnancy in men in a non-confrontational way to elicit his beliefs. We questioned the patient about his belief in men becoming pregnant, given that we typically only witness women conceiving. He stated that he had seen a pregnant male in the media, and so it wasn’t surprising to him. Several days later, the discussion of obtaining a pregnancy test was brought up, to which the patient agreed. When given the negative results of his pregnancy test, he began to cry, self-described “tears of joy,” because he “no longer has to eat for two.” It is unclear if this implies that he believes that he was never pregnant or was once pregnant and is no longer. Nevertheless, the use of a pregnancy test with the patient’s consent helped to extinguish the delusion from which he was suffering. This strategy of gentle investigation of the beliefs around the physical possibility of being pregnant, combined with the use of a real pregnancy test to dissolve the delusion of pregnancy, can be of utility to future clinicians who may encounter similar presentations.

## Conclusions

This case highlights the rare presentation of an older male suffering from the delusion of pregnancy. It shares commonalities with numerous other cases reported in the literature of delusion of pregnancy in males arising after a homosexual encounter, but is unique for the age of the patient and the lack of gender dysphoria. The use of a pregnancy test for reality orientation in patients with delusions of pregnancy is a low-risk and low-cost method to attempt to dispel the delusion. This method can be helpful to future clinicians who may find themselves with patients suffering from delusions of a similar nature and could warrant formal evaluation.
